# Comparison of Materials for Posterior Facial Frame Injection: Hyaluronic Acid or Polycaprolactone, a Case Report

**DOI:** 10.1111/jocd.70315

**Published:** 2025-07-30

**Authors:** Liping Hu, Xi Chen, Guangpeng Liu

**Affiliations:** ^1^ Department of Dermatology Weizhuan Medical Aesthetics Clinic Shanghai China; ^2^ Department of Dermatology Jinshi Medical Aesthetics Clinic Shanghai China; ^3^ Department of Plastic and Reconstructive Surgery Shanghai Tenth People's Hospital, Tongji University School of Medicine Shanghai China

**Keywords:** aging face, face lift, hyaluronic acid

To the Editor,

We reported last year in Journal of Cosmetic Dermatology about an injection method to improve the posterior facial frame (PFF) titled “Area four technique: An effective injection approach to the posterior facial frame esthetic treatment with injectable hyaluronic acid fillers” [1]. Due to the superior effect on facial lifting and contour improvement, this technology attracted wide attention in China.

More and more patients pay attention to the effect of facial improvement and tightening after PFF injection, making us think about what material is the most appropriate to place in the PFF area. The facial lifting of PFF injection is due to the mechanical support of the fascia by the filling. However, with the degradation of hyaluronic acid (HA) in the body, the mechanical support ability of HA gradually weakens and cannot effectively and sustainably support the fascia or ligaments. As a result, continued facial sagging and other complications such as overfilled syndrome occur [2].

In 2009, a novel polycaprolactone (PCL) filler (Ellansé, Sinclair Pharma, UK) obtained the CE Marking and was introduced in Europe and many of the leading countries in esthetics worldwide [3]. The novel filler made up of polycaprolactone (PCL, 30% by volume) and carboxymethyl cellulose (CMC, 70% by volume) owns a dual effect, an immediate effect and a long‐term effect [3]. The immediate effect is related to the CMC gel by the filling capacity of the injected volume and the highly hygroscopic properties of CMC. The CMC gel is resorbed in 2–3 months. The followed sustained effect depends on the collagen production by the evenly distributed PCL microspheres embedded with collagen fibers interacting with the cellular environment [4]. The collagen deposit that adheres closely to the surrounding tissue leads to the prolongation of the facial improvement and tightening.

To observe the clinical difference between PCL and HA in facial enhancement, we injected 6 mL of PCL (Ellansé, Sinclair Pharma, UK) and 6 mL of HA (Vmonalisa, Dentium, Korea) into the right face (PCL sdie) and the left face (HA side) of the subject according to Area four technique, and recorded photos immediately, 45, 90, and 180 days after surgery (Figure 1).

**FIGURE 1:**
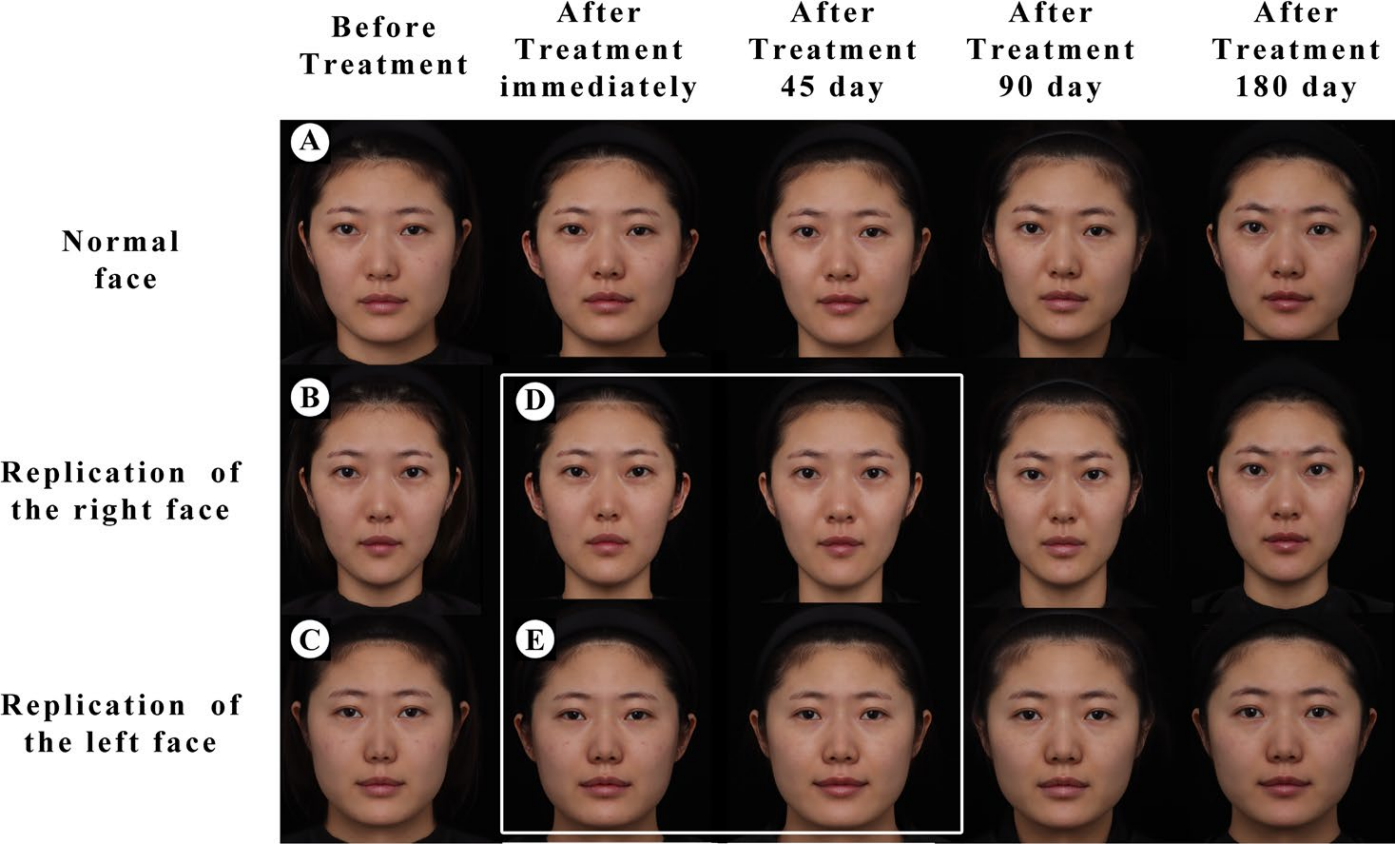
The two sides of the face appeared as an uncoordinated phenomenon after injection during the 180 days of follow‐up (A). The left and right lateral faces were cut and re‐synthesized (B, C). The lifting effect of the right face injected with PCL remained stable for 180 days (D). The lifting effect of the left face injected with HA was weakened on day 45 (E).

During the 180 days of follow‐up, both sides of the face were improved immediately after the surgery. However, the two sides of the face appeared as an uncoordinated phenomenon gradually (Figure 1A). Because the facial asymmetry of the subject affected the observation of treatment, the left and right lateral faces were cut and re‐synthesized (Figure 1B,C). Therefore, we can intuitively observe that the lifting effect of the right face remained stable for 180 days, while the lifting effect of the left face was weakened on day 45 (Figure 1D,E). In addition, the subject experienced the Spring Festival holiday on postoperative day 180 and gained increased weight. However, despite the increase in the right soft tissue volume of the face, the droop is not obvious.

## 1 Discussion

In a 2020 study, it was proposed that HA for ligament treatment could achieve facial lifting [5]. In our previous work, we also combined classic articles to illustrate the facial lifting effect of HA for the true ligament and superficial muscular aponeurotic system (SMAS) [6]. The key point of the technique is the support of the filler for the ligament structure, which lifts the sagging tissue.

However, it is well known that HA gradually loses its support as it metabolizes in the body. As a result, tissues that have been lifted become saggy again. PCL is an effective collagen stimulant. After PCL was implanted into the human body, it acts as a collagen stimulator to continuously stimulate the regeneration of collagen. It mainly stimulates the formation of type I collagen. The scaffold formed by the new collagen can play a certain volume effect in the long term. Moreover, the type I collagen stimulated by PCL provides stronger and more stable support in the body, and the final degradation products of PCL are carbon dioxide and water [7, 8, 9, 10].

There are no studies on whether to choose regenerative materials such as PCL or inert filling materials such as HA for facial lift injections. However, physicians can get a lot of patient feedback and infer from the characteristics of the injection material. Therefore, we carried out this clinical follow‐up study of observation for half a year. Although the number of subjects did not meet the statistical criteria, it still has reference significance for clinical medical treatment. Based on the current observation study, we believe that PCL is better than HA for the injection of facial lifting points. In the future, we will continue to study the histological changes of the regenerated materials in vivo and the physical properties in vitro, to provide more basic research evidence for clinicians.

## Author Contributions

X.C. designed the study and wrote the paper and edited the pictures. L.H. and G.L. provided the injection site and reviewed the article.

## Consent

Subject was signed written consent forms and known detailed information about the treatment. All operations involving human participants are approved by institutional ethics committees and conform to the ethical standards of institutions and national research committees and the ethical standards of the World Medical Association Declaration of Helsinki (June 1964).

## Conflicts of Interest

The authors declare no conflicts of interest.

## Data Availability

The data that support the findings of this study are available from the corresponding author upon reasonable request.
